# Tauopathic Changes in the Striatum of A53T α-Synuclein Mutant Mouse Model of Parkinson's Disease

**DOI:** 10.1371/journal.pone.0017953

**Published:** 2011-03-21

**Authors:** Jonathan Wills, Joel Credle, Thomas Haggerty, Jae-Hoon Lee, Adam W. Oaks, Anita Sidhu

**Affiliations:** Department of Biochemistry, Molecular and Cellular Biology, Georgetown University, Washington, D.C., United States of America; The Mental Health Research Institute of Victoria, Australia

## Abstract

Tauopathic pathways lead to degenerative changes in Alzheimer's disease and there is evidence that they are also involved in the neurodegenerative pathology of Parkinson's disease [PD]. We have examined tauopathic changes in striatum of the α-synuclein (α-Syn) A53T mutant mouse. Elevated levels of α-Syn were observed in striatum of the adult A53T α-Syn mice. This was accompanied by increases in hyperphosphorylated Tau [p-Tau], phosphorylated at Ser202, Ser262 and Ser396/404, which are the same toxic sites also seen in Alzheimer's disease. There was an increase in active p-GSK-3β, hyperphosphorylated at Tyr216, a major and primary kinase known to phosphorylate Tau at multiple sites. The sites of hyperphosphorylation of Tau in the A53T mutant mice were similar to those seen in post-mortem striata from PD patients, attesting to their pathophysiological relevance. Increases in p-Tau were not due to alterations on protein phosphatases in either A53T mice or in human PD, suggesting lack of involvement of these proteins in tauopathy. Extraction of striata with Triton X-100 showed large increases in oligomeric forms of α-Syn suggesting that α-Syn had formed aggregates the mutant mice. In addition, increased levels of p-GSK-3β and pSer396/404 were also found associated with aggregated α-Syn. Differential solubilization to measure protein binding to cytoskeletal proteins demonstrated that p-Tau in the A53T mutant mouse were unbound to cytoskeletal proteins, consistent with dissociation of p-Tau from the microtubules upon hyperphosphorylation. Interestingly, α-Syn remained tightly bound to the cytoskeleton, while p-GSK-3β was seen in the cytoskeleton-free fractions. Immunohistochemical studies showed that α-Syn, pSer396/404 Tau and p-GSK-3β co-localized with one another and was aggregated and accumulated into large inclusion bodies, leading to cell death of Substantia nigral neurons. Together, these data demonstrate an elevated state of tauopathy in striata of the A53T α-Syn mutant mice, suggesting that tauopathy is a common feature of synucleinopathies.

## Introduction

α-Synuclein (α-Syn) is expressed in the brain and is highly enriched in presynaptic nigrostriatal nerve terminals, where its chief physiological function may be the regulation of synaptic levels of dopamine and other monoamines through modulation of the monoamine transporters [1]. Overexpression of α-Syn, through its gene duplication and triplication, is linked to idiopathic Parkinson's disease (PD), while its A30P and A53T mutants cause the autosomal dominant forms of familial PD [2]–[4]. In pathological states, α-Syn becomes misfolded, aggregates and accumulates into neuronal inclusion bodies and Lewy bodies (LBs), hallmarks of PD and other synucleinopathies [2], [5]–[6]. Post-mortem immunohistochemical studies show the presence of hyperphosphorylated Tau (p-Tau), a protein normally linked to the genesis of Alzheimer's disease (AD), co-existing with α-Syn aggregates in the same neurons in PD and other synucleinopathies [7]–[14]. Conversely, in tauopathies such as AD, elevated levels of α-Syn have been found [15]–[20], along with LBs [15], [21], [22]. More recently, we have shown in human post-mortem PD striata that in addition to elevated levels of insoluble aggregates of α-Syn, p-Tau levels were also increased [23]. Thus, we found increases in p-Tau hyperphosphorylated at Ser202, Ser262 and Ser396/404; these are the same sites that are also primarily hyperphosphorylated in AD, leading to pathological changes. In addition, we also found increases in levels of active GSK-3β [p-GSK-3β, hyperphosphorylated at Tyr216], the kinase known to hyperphosphorylate Tau at the above mentioned sites. Together, these data suggest that synucleinopathies and tauopathies may actually be overlapping or interacting neurodegenerative diseases, rather than distinct entities.

Using the MPTP mouse neurotoxin model of PD, as well as cellular models of PD, we had previously demonstrated that increases in α-Syn can initiate and sustain Tau hyperphosphorylation both *in vivo* and *in vitro* [24]–[27]. The hyperphosphorylation of Tau in our studies was absolutely dependent on the presence of α-Syn, as indexed by lack of p-Tau formation in either MPTP-treated α-Syn−/− mice, or in toxin-treated neuronal cells lacking α-Syn [24], [27]. The underlying reason for the mandatory requirement for α-Syn in formation of p-Tau is not clear, but our studies suggest that α-Syn can recruit and activate GSK-3β, via autophosphorylation at Tyr216 [26]–[27], suggesting a tight interaction between α-Syn and p-GSK-3β is necessary for Tau hyperphosphorylation. Moreover, when incubated together *in vitro*, α-Syn may serve as a seed, accelerating the aggregation of Tau [12].

We undertook the current investigation to analyze the state of tauopathy in a transgenic mouse model of PD, the hemizygous transgenic mice expressing the human α-Syn A53T mutant [28]. Tauopathic changes were observed in the striata of the A53T α-Syn transgenic mice. Moreover, such changes were similar to those found in striata of human PD patients. The A53T α-Syn was aggregated, along with pSer396/404 Tau and p-GSK-3β. α-Syn was found to be bound to the cytoskeleton, while pSer202, pSer262 and pSer396/404 Tau and p-GSK-3β were present in cytoskeletal-free fraction. Immunohistochemical studies showed that α-Syn, p-Tau and p-GSK-3β co-localized with one another and were accumulated into inclusion bodies.

Together, our studies indicate an elevated state of tauopathy in striata of the A53T α-Syn mutant mice, reminiscent of that seen in PD striata, lending further credence to our conclusion that tauopathy is also a common feature of synucleinopathies.

## Results

### α-Syn, p-Tau and GSK-3β protein expression levels in A53T mutant mouse

**Figure 1:**
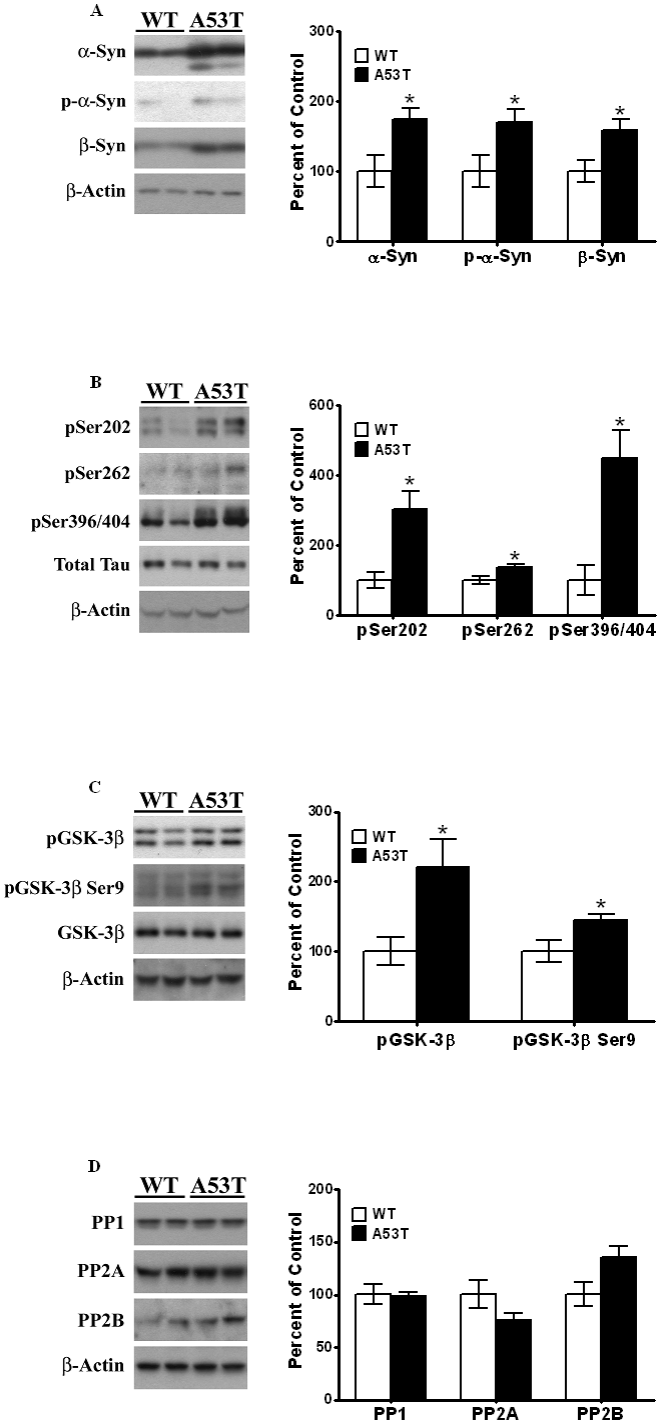
Elevated levels of α-Syn, p-Tau and p-GSK-3β in striata of α-Syn A53T mutant mice. Striata from wild type non-transgenic mice and A53T α-Syn mutant transgenic mice, 8 months of age, were dissected and homogenized in RIPA buffer, and analyzed by Western blots, as described under “Materials and Methods”. After exposure to initial antibodies, blots were stripped and probed for other proteins. The blots show representative gels while the bar graphs are composites summarized from all animals (*n* = 4–9). (A) α-Syn and p-α-Syn were expressed relative to β-actin used as a loading control. (B). p-Tau was probed using antibodies specific for pSer202, pSer262 and pSer396/404, and expressed relative to total Tau used as loading control. (C). p-GSK-3β levels were probed using antibodies which recognize phosphorylation at Tyr216, and expressed relative to total GSK-3β. (D) PP1, PP2A and PP2B levels were probed using specific antibodies and normalized to β-actin. Asterisks (*) indicate values significantly different from wild-type animals (*P*<0.05). Student's t-test was performed for all data.

PIPES/SDS extracts of striatum from A53T α-Syn mutant mice were recombined to obtain total extracts, as described in Methods. and these extracts were examined by Western blots to assess changes in proteins we have previously shown to be involved in the tauopathic pathway in different PD models [24]–[27]. The expression levels of α-Syn [M_r_ 17 kDa] were significantly [*P<*0.05] increased by 75% in the A53T mutant mice [*n* = 4], as compared to litter-mate, age-matched wild-type animals [*n* = 4, Fig. 1A]. In addition, an increase [of 70%, n = 4, *P<*0.05] in p-α-Syn [M_r_ 16 kDa], detected by a phospho-specific antibody was also detected in A53T mutant mice, as compared to wild type litter-mate controls [Fig. 1A]. We also examined β-Syn levels [M_r_ 16∼kDa] and found increased [58%, n = 4, *P<*0.05] levels of this protein in A53T mutant mice.

The overexpression of A53T α-Syn and β-Syn was accompanied by an increase in p-Tau levels hyperphosphorylated at different epitopes [Fig. 1B]. Thus, there was a significant [*P<*0.05] increase of 204% in p-Tau hyperphosphorylated at Ser202 [M_r_ 55 kDa; n = 6], along with a significant [*P<*0.05] increase of 36% in pSer262 [M_r_ 60 kDa] in mutant mice [*n* = 6], as compared to wild-type mice [n = 9]. Similarly, p-Tau hyperphosphorylated at Ser396/404 [M_r_ 55 kDa], was also significantly [p<0.05] increased by 349% in mutant mice as compared to wild-type [*n* = 6 and 9, respectively].

We next measured changes in p-GSK-3β, hyperphosphorylated at Tyr216, a major kinase known to phosphorylate Tau [Fig. 1C]. The antibody against p-GSK-3β also recognizes p-GSK-3α, detectable as a band of M_r_ ∼50 kDa visible above p-GSK-3β; in our studies, only the lower band, corresponding to p-GSK-3β [M_r_ of 46 kDa] was used to calculate for levels of this protein. We found significant [*P<*0.05] increases of 128% in the levels of p-GSK-3β in mutant mice relative to wild-type animals [*n* = 4 and 4, respectively]. Moreover, a significant increase [of 45%] in levels of the inactive form of GSK-3β, hyperphosphorylated at Ser9 [M_r_ 46 kDa], was also seen in striatum of the A53T mutant mouse. No significant changes were observed in β-actin [M_r_ 43 kDa], total Tau [bands with M_r_ of 52–68 kDa] or total GSK-3β [M_r_ 46 kDa] between the two groups of mice. Together, these data indicate the existence of an elevated state of tauopathy in striata of the A53T α-Syn mutant mice.

To eliminate the possibility that decreases in protein phosphatases [PP] lead to an increase in p-Tau levels, we also examined the levels of the major protein phosphatases found in brain, PP1 and PP2 [Fig. 1D]. There were no significant [*P>*0.05] differences in the expression levels of PP1, PP2A or PP2B [M_r_ of 38, 62 and 55 kDa, respectively] between A53T or wild type controls, [n = 6], suggesting that increases in p-Tau were not due to decreases in protein phosphatases.

### Increase in p-Tau and p-GSK-3β in striata of postmortem PD brains

**Figure 2:**
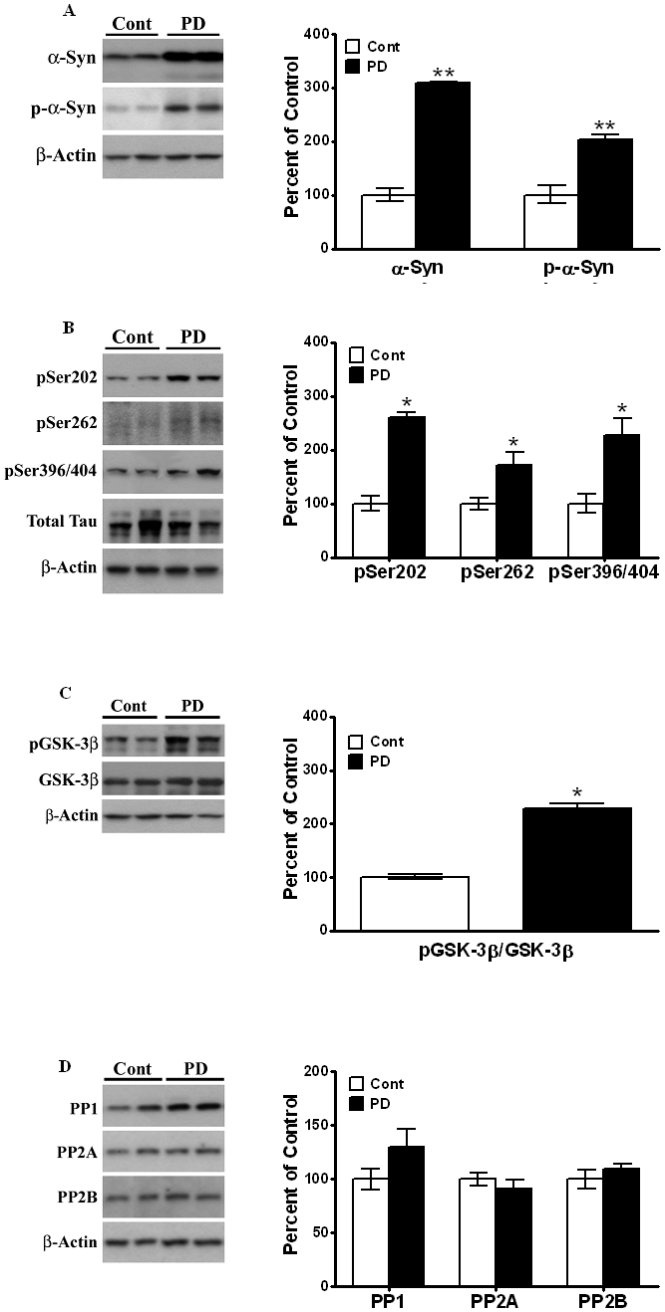
Postmortem PD brains show increased levels of α-Syn, p-GSK-3β and p-Tau at phosphorylation sites Ser202, Ser262 and Ser396/404 similar to striatum in the 8 month-old A53T mice. Postmortem control and PD brain lysates were prepared and immunoblots probed for (A) α-synuclein and p-α-synuclein, (B) phospho-specific Tau epitopes at Ser202, Ser262 and Ser396/404 and (C) p-GSK-3β. (D) PP1, PP2A and PP2B levels were probed using specific antibodies and normalized to β-actin. Representative immunoblots from postmortem samples are shown. β-actin levels were measured as loading controls. Increases in the level of α-synuclein and are normalized to β-actin and data are expressed as a percent increase relative to amounts present in control, non-diseased brains (100%). Phospho-specific Tau and p-GSK-3β are expressed as a percent increase with respect to non-diseased controls (100%) after normalization to total Tau and GSK-3β, respectively. Values are mean ± SEM. Asterisks (*) indicate values significantly different from age-matched control postmortem striata (p<0.05). Student's t-test was performed for all data.

In order to ascertain whether tauopathic changes in the A53T mice resemble that seen in humans, we examined tauopathy in striata from postmortem brains of PD patients. α-Syn levels were measured in postmortem striata of 6 control subjects [Fig. 2A] and from 6 patients clinically diagnosed with PD, as previously described in Methods. In the PD patient samples tested, expression levels of α-Syn were increased [208%, *P*<0.05], as compared to age-matched controls. When we measured changes in p-α-Syn in PD, we found significant [*P*<0.05] increases in the phosphorylated protein [of 104%, n = 7] in PD striata compared to non-diseased controls.

We next examined p-Tau levels in the striata of controls and PD patients [Fig. 2B]. There were no significant changes in total Tau levels between control subjects and PD patients. However, significant increases were observed for all p-Tau epitopes measured. We found increases of 161% [*P*<0.05] in the levels of Tau hyperphosphorylated at pSer202 in PD striata compared to striata from control subjects [Fig. 2B]. Hyperphosphorylation of Tau at pSer262 was increased by 72% [*P*<0.05] in striata of PD patients relative to controls. Tau phosphorylation at pSer396/404 was increased by 128% [*P*<0.05] in PD striata relative to age-matched controls [Fig. 2B]. Thus, significant increases in Tau hyperphosphorylation are present at the same three epitopes in human PD as those detected in A53T mutant mice.

We also analyzed p-GSK-3β levels in control and PD human samples and found an increase of 128% [*P*<0.05] in PD brains compared to age-matched controls [Fig. 2C].Thus, while the absolute values of the increases vary between human PD and the A53T mutant mouse, there is nonetheless an analogous pattern of increase in α-Syn, p-Tau proteins, and p-GSK-3β, suggesting that this animal model of PD shows similar tauopathy as human PD.

We next examined changes in PP levels in striatum of PD [Fig. 2D]. There were no significant changes in levels of either PP1, PP2A or PP2B between PD and control striata, similar to our findings in A53T transgenic and wild type, again suggesting that alterations in protein phosphatases do not contribute to increases in p-Tau levels.

### Triton X-100 solubilization of striatal proteins

**Figure 3:**
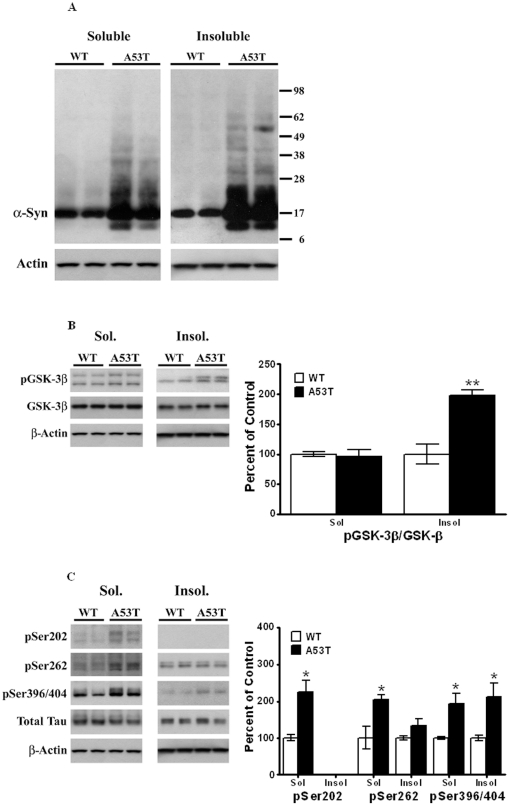
Triton X-100 solubilization of striatal lysates from A53T α-Syn mutant and age-matched control animals. Striatal lysates from A53T α-Syn mutant mice and control, non-Tg mice [4 animals per group] were extracted with Triton X-100 as described in Methods. Proteins in Triton X-100-soluble and Triton X-100-insoluble fractions were measured by Western blots. Blots show representative gels while the bar graphs are composites summarized from all animals, expressed as percent of control, non-Tg mice. (A) β-actin was added as a loading control. (B) p-GSK-3β was normalized to GSK-3β from within each fraction on the blot. (C) pSer202, pSer262 and PSer396/404 were all normalized to total Tau in each fraction, and β-actin was added as a loading control. *, *P<*0.05; **, *P<*0.01 and indicate values significantly different from wild-type animals. Student's t-test was performed for all data.

Previous studies have shown a differential solubility of α-Syn in 1% Triton X-100, whereby aggregates of α-Syn were found to segregate in Triton X-100-insoluble fractions upon centrifugation [30]. To test whether aggregates of α-Syn were present in striata of the A53T mutant mouse, striatal tissues were extracted in Triton X-100, as described under Methods, and this protein was examined in both soluble and insoluble fractions [Fig. 3A]. Compared to wild type non-transgenic animals [*n* = 4], α-Syn isolated from the A53T transgenic mice were increased in both Triton X-100 soluble and insoluble extracts. Whereas the majority of the α-Syn in Triton X-100-soluble extracts from wild type animals was present as a 17 kDa band, in the transgenic mice additional bands with M_r_ of ∼24 and 35 kDa were present, along with faint bands >38 kDa and a band below 17 kDa, with M_r_ of ∼12 kDa. In the Triton X-100-insoluble fraction, a single band corresponding to the monomeric form of the protein was observed in the wild type animal [Fig. 3A]. In the transgenic A53T mice, multiple bands were seen. Thus, strong bands of M_r_ of 12 and 24 kDa were seen; a 34 kDa band, likely representing a dimeric form of the 17 kDa species was seen; a faint band at ∼40 kDa was seen. Finally, a prominent band at ∼52 kDa was seen, which may represent a trimeric form of the 17 kDa monomer.

**Figure 4:**
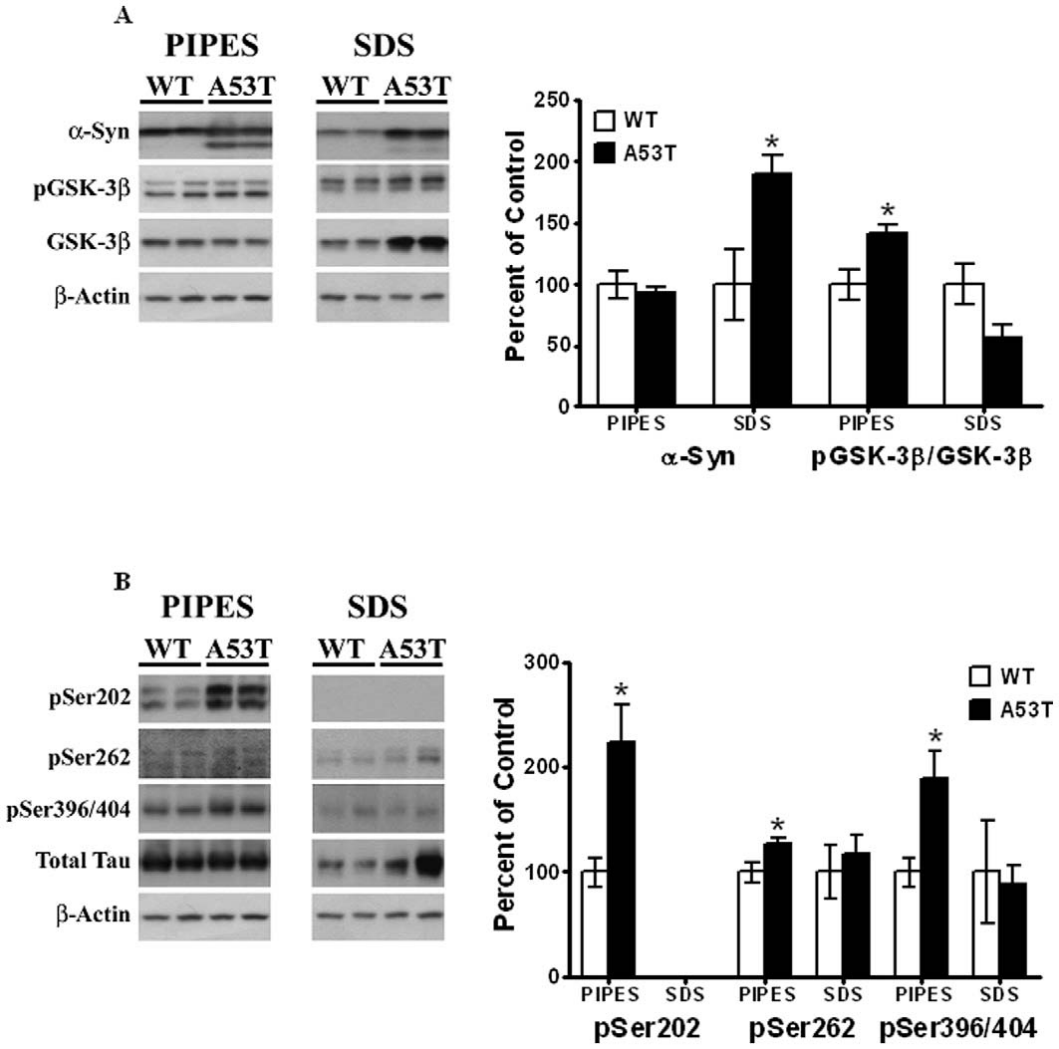
Binding of striatal proteins from A53T α-Syn mutant and wild-type non-Tg mice to cytoskeleton. Lysates from striata of mutant and non-Tg mice [*n* = 4 per group] were extracted in PIPES buffer and SDS buffer to obtain cytoskeleton-free and cytoskeleton-bound fractions, as described in Methods. (A) α-Syn and p-GSK-3β were normalized to β-actin and GSK-3β, respectively, while (B) pSer396/404 was normalized to total Tau. Results were expressed as percent of control non-Tg mice. Blots show representative gels while the bar graphs are composites summarized from all animals. *, *P<*0.05; **, *P*<0.01 and indicate values significantly different from wild-type animals. Student's t-test was performed for all data.

The 14 kDa band is noteworthy in that it is only seen in the A53T mutant and not in the wild type mice [see Figs. 1A, 3A and 4A]. Although it remains to be confirmed, this polypeptide may represent either a truncated form of the full length α-Syn or, alternatively, a degraded form of the 17 kDa protein. Regardless, the results of Triton X-100 extractions suggests that this protein aggregates capable of forming dimers of 28 kDa and trimers of ∼40 kDa [see Fig. 3A].

When similar studies were conducted with p-GSK-3β [Fig. 3B], we found no significant differences in the levels of p-GSK-3β in the soluble fraction, whereas the levels of p-GSK-3β present in the Triton X-100-insoluble fractions were increased by 98% [*P<*0.01, *n* = *4*]. This finding was surprising, since p-GSK-3β is a soluble protein, and its presence in the insoluble fraction suggests that it has become aggregated and/or that a co-interaction of the kinase with α-Syn must exist, causing it to co-segregate with α-Syn in the insoluble fraction. Indeed, in earlier studies, using a MPTP-treated mouse model which develops tauopathy, we have shown that activated p-GSK-3β co-immunoprecipitates with α-Syn and with pSer396/404 Tau [24].

When we examined p-Tau levels in the Triton X-100 soluble and insoluble fractions [Fig. 3C], we found large significant increases [124%, *P*<0.05, *n* = 4] in pSer202 levels in the soluble fraction in the A53T mice; this epitope was not detected in the Triton X-100 insoluble fraction for either the transgenic or wild type mouse. Significant increases were also seen for pSer262 in the Triton X-100 soluble fraction [105%, *P*<0.05, *n* = 4] in A53T mice. Although a small increase in pSer262 was seen in Triton X-100-insoluble fractions [Fig. 3C], this increase was not significant [*P*>0.05]. In the Triton X-100-soluble fractions of A53T mice, pSer396/404 Tau levels were significantly [*P<*0.05, *n* = 4] increased by 93%, whereas in the Triton X-100-insoluble fraction it was increased by 112% [*P*<0.05, *n* = 4] compared to wild type animals. The presence of high levels of pSer396/404 in the Triton X-100-insoluble fraction suggests its aggregation and/or interaction with aggregated α-Syn.

### Interactions of α-Syn, p-Tau epitopes and p-GSK-3β with the cytoskeleton

Both α-Syn and Tau are microtubule binding proteins; in addition, we have shown that α-Syn can also bind to the actin cytoskeleton [31]. Upon hyperphosphorylation, p-Tau is known to dissociate from microtubules. We therefore felt it worthwhile to analyze how α-Syn and p-Tau interact with the cytoskeleton proteins in the striatum of the A53T mutant mice. To conduct these studies, striatal tissue was differentially extracted in PIPES and SDS buffer, as described in Methods, to isolate cytoskeleton-free and cytoskeleton-bound proteins, respectively.

Similar levels of α-Syn were seen in the PIPES-extracted cytoskeleton-free fractions of A53T mice, as compared to wild-type controls [Fig. 4A]. Interestingly, there was a large [90%] and significant [*P<*0.05, *n* = 4] increase in α-Syn levels observed in the SDS-extracted cytoskeleton-bound fraction. In parallel studies conducted with p-GSK-3β [Fig. 4A], increased levels [42%, *P<*0.05, *n* = 4] of this protein were observed in the cytoskeleton-free fraction of A53T mice as compared to insignificant decreases in the cytoskeleton-bound fraction. This finding is entirely consistent with the fact that neither p-GSK-3β nor GSK-3β is known to directly interact with cytoskeleton proteins.

When we examined p-Tau interactions with the cytoskeleton [Fig. 4B]. pSer202 was exclusively detected only in the PIPES-extracted, cytoskeleton-free fractions, and significant increases in pSer202 were seen in these fractions in A53T mice [123%, n = 4, *P*<0.05]. Similarly, increases in pSer262 were seen in cytoskeleton-free fractions in A53T mice [27%, n = 4, *P*<0.05]; a small amount of pSer262 remained associated with the cytoskeleton fraction, but there were no significant differences between wild type and mutant mice. Finally for pSer396/404, increases in this protein were seen in only in cytoskeleton-free fractions in extracts from mutant mice [89%, n = 4, *P*<0.05], while no differences were detected between these mice and wild type mice. These combined data suggest that upon hyperphosphorylation, Tau dissociates from the cytoskeleton fraction.

### Immunohistochemical co-localization and distribution in striatum

**Figure 5:**
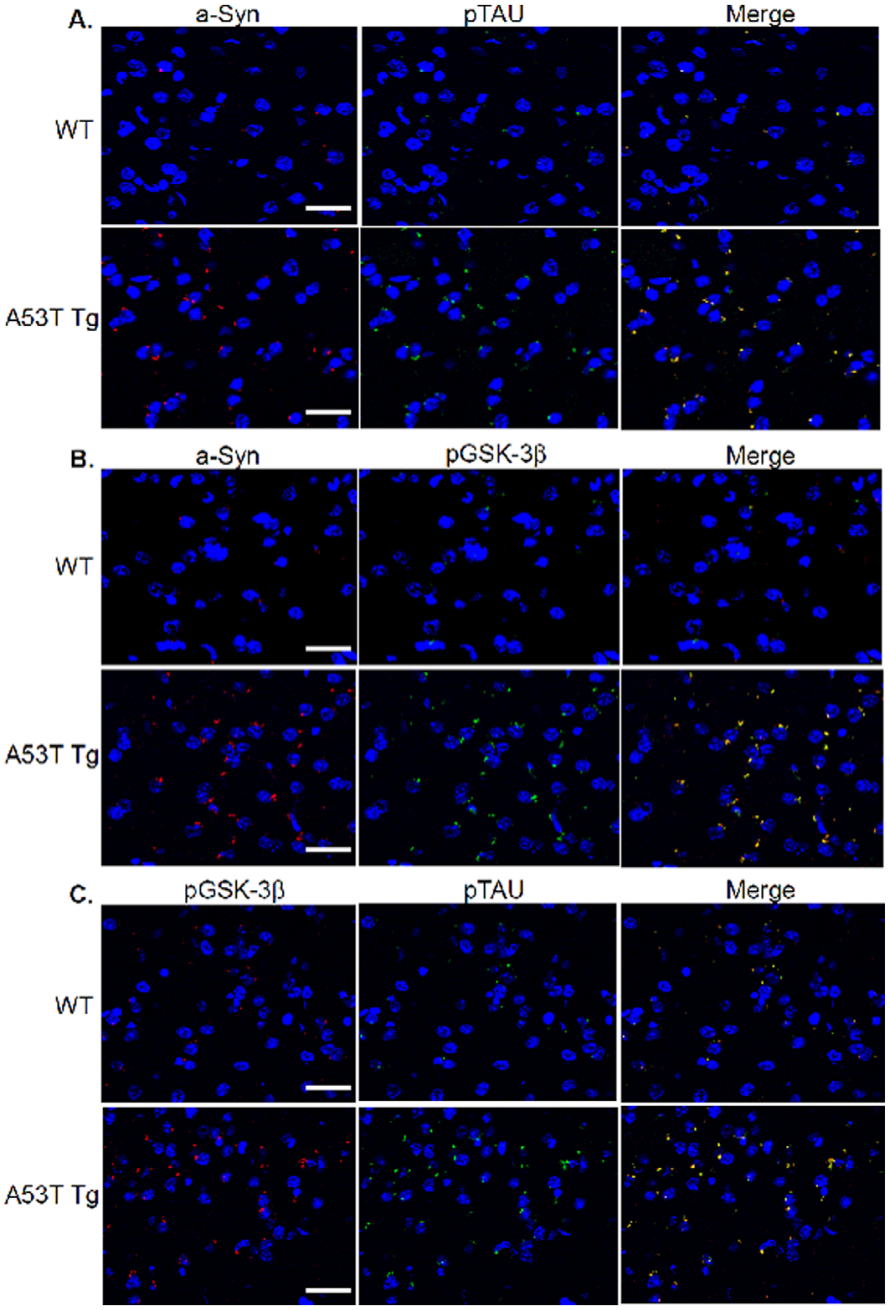
Immunohistochemical co-localization and distribution in A53T α-Syn and wildtype mice. Right panels constitute merged image of left panels. (A). Striatum stained with α-Syn (Red) and PHF-1 Tau (Green) with DAPI as merged, and with single antibody(s). (B) Striatum stained with α-Syn (Red) and p-GSK3β (Green) with DAPI as merged, and with single antibody(s). (C) Striatum stained with p-GSK-3β (Red) and PHF-1 (Green) with DAPI as merged, and with single antibody(s). Scale bar: 70 µm.

The aggregation and co-localization of α-Syn with pGSK-3β and p-Tau was analyzed by immunohistochemical [IHC] staining of the striatum of A53T α-Syn mutant mice and compared to age-matched wild-type mice [Fig. 5A–C]. An overall total increase in levels of α-Syn, pGSK-3β, and p-Tau was seen in the striatum of A53T α-Syn mice as compared to wild-type control striatum. Dual staining was performed to analyze the co-localization of the following protein combinations: α-Syn:p-Tau, α-Syn:p-GSK-3β and p-Tau:p-GSK-3β. Each protein combination co-localized together in a single large aggregate formation in the transgenic striatum, as opposed to the multiple small puncta and more diffuse cytoplasmic distribution seen in the WT striatum. This large aggregate seen in the Tg striatum is reminiscent of pathogenic Lewy bodies in shape, size, and cellular location.

**Figure 6:**
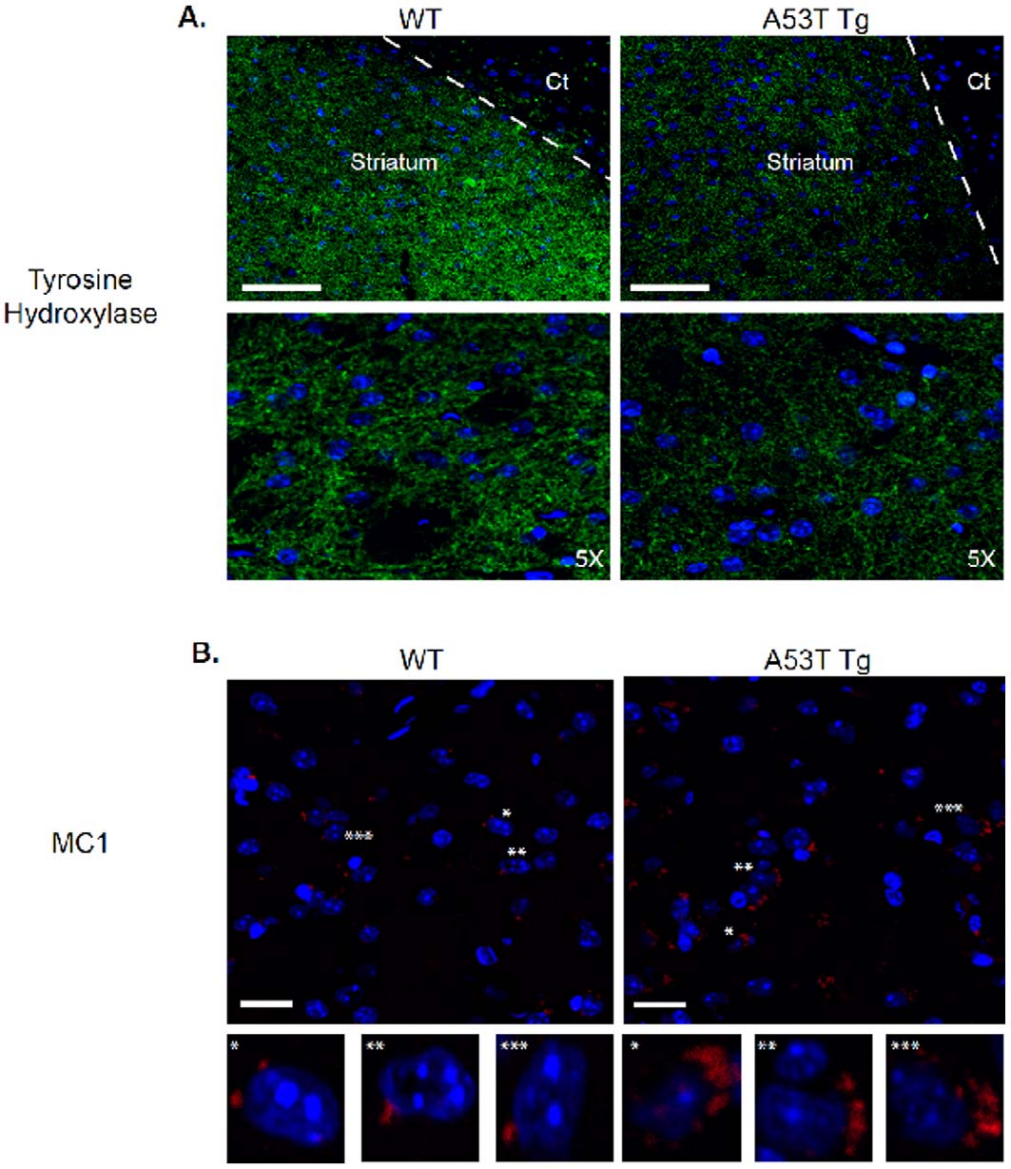
TH and MC1 immunostaining in A53T α-Syn and wildtype mice. Immunohistochemical staining of tyrosine hydroxylase (TH, A) or p-Tau using the MC1 antibody (B) was conducted as described in Methods, using DAPI to stain nuclei. Slides are shown at lowest magnification (upper panels) to highest magnification (lower panels). (A). Dashed line represents demarcation of striatum with cortex (Cx). Scale bar: 70 µm. (B). Asterisks indicate highlighted individual cells shown at higher magnification in lower panels. Scale Bar: 10 µm.

Further IHC analysis examined the temporal loss of TH positive cells in the striatum when comparing the striatal region in A53T and wildtype mice [Fig. 6A]. A definitive loss of TH positive neurons is observed in the striatum of Tg mice when compared to WT. The TH antibody also shows a clear distinction between the abundant TH-positive neuron containing striatum versus the absence of such TH positive neurons in the cortex. To observe a conformational aggregation-prone version of Tau, the MC1 antibody was used to stain striatal sections [Fig. 6B]. In the striatum of Tg mice, large aggregates are present, in a peri-nuclear fashion, with only small infrequent puncta seen in the WT striatum.

### Immunohistochemical co-localization and cell loss of Substantia nigra

**Figure 7:**
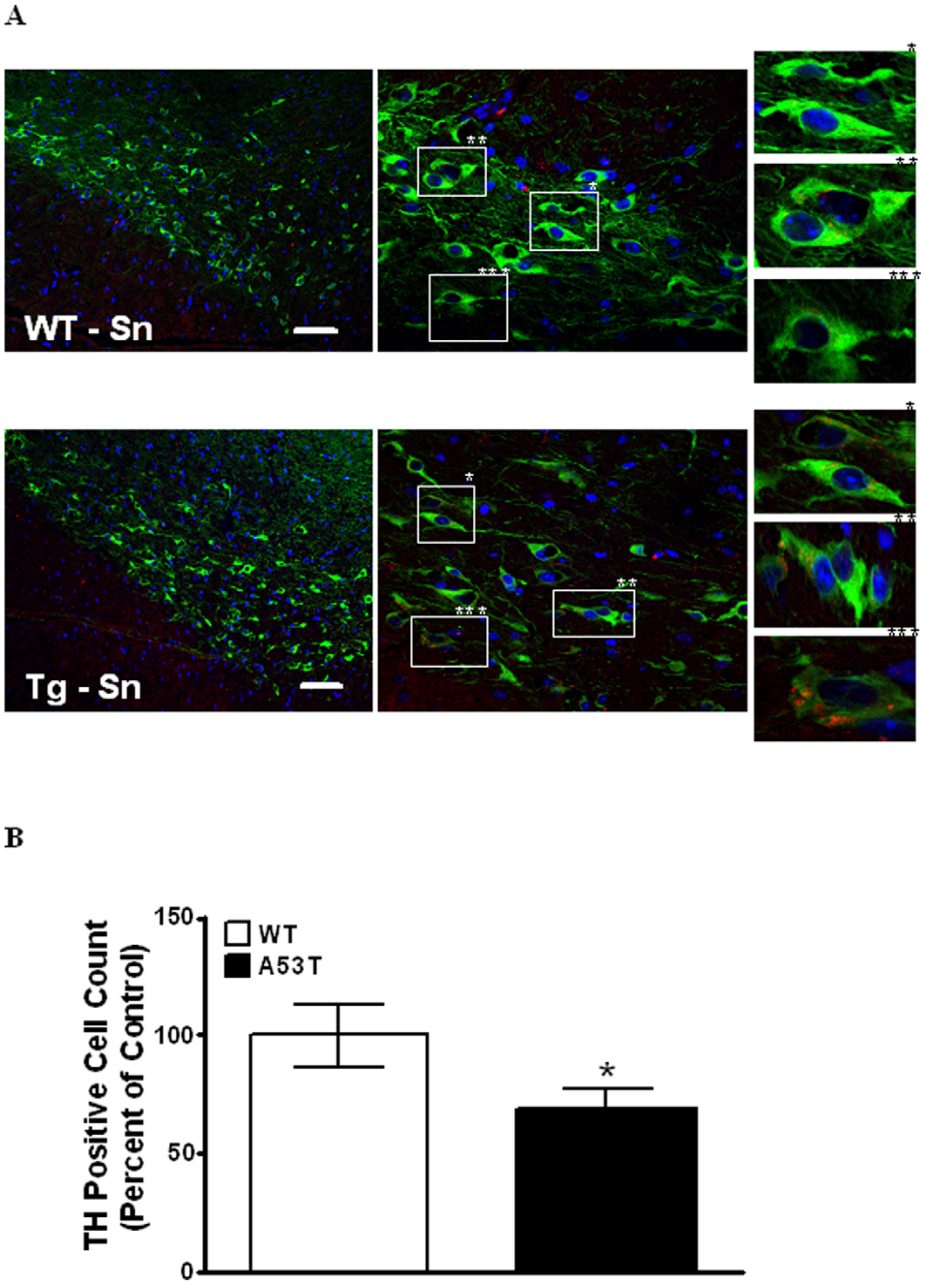
Immunohistochemical co-localization and counting of dopaminergic neurons in *Substantia nigra*. (A). Double immunostaining of *Substantia nigra* stained for α-Syn (Red) and TH (Green) with DAPI. Panels left to right: 20X, 60X, with white boxes highlighted on left panels indicate areas shown at higher magnification in far right panel(s) in wild type and A53T transgenic mouse brain. (B). Staining and counting of TH (Green) positive dopaminergic neuronal cells within the *Substantia nigra*. Panels left to right: 20X (large panels), 60X (small panels), with white boxes and asterisks highlighted on left panels indicating areas shown at higher magnification in far right panel(s) in wild type and A53T transgenic mouse brain. Values are mean of (n = 3 mice) Six *Substantia nigra* sections per WT and Tg mouse. P<0.05. Scale bar 10 u*M*. All images were taken on an Olympus Fluoview FV300 using lenses: 20X N.A. 1.0, 60X N.A. 1.4. Images were processed in imageJ v1.44 (NIH) using the cell counter plug-in for automated cell counting.

We also examined the Substania nigra of both A53T transgenic and wild mice, in order to asses the effects of tauopathy on neurons in this region of the brain. Fig. 7A shows representative images of the distribution and co-localization of α-Syn in TH positive neurons of the *Substantia nigra* of wild type and A53T transgenic mice. White Boxes (Fig. 7A middle panel) highlight single dopaminergic cells in far right panels which show the presence or absence of α-Syn in TH positive cells in A53T transgenic and wild type mice, respectively. A higher number of TH positive cells have increases in the relative amounts of α-Syn detected in A53T transgenic versus WT mice. This increase in the amounts of α-Syn co-localizing with TH positive cells, being greater in A53T transgenic *Substantia nigra* versus wild type, was quantitatively assessed and verified using intensity correlation analysis [32]. Dopaminergic cell loss was quantified using methods described in legend to Fig. 7 [Fig. 7B]. The A53T transgenic mouse shows a significant [*P*<0.05, n = 3] decrease of 30.7% in TH positive DA neurons of the *Substantia nigra* versus wild type. Such partial losses of DA neurons that we see in the *Substantia nigra* of the A53T transgenic model, have also been previously described in genetic, and toxin based mouse models of PD [33], [34].

## Discussion

We describe for the first time the tauopathic changes occurring in the striatum of the A53T α-Syn mutant hemizygous mouse model of PD. We have previously demonstrated the hyperphosphorylation of Tau *in vivo* in the MPTP-induced neurotoxin mouse model of parkinsonism [24], [27] and *in vitro* in MPP^+^-treated primary mesencephalic neurons [26], in SH-SY5Y neuronal cells [24]–[25], as well as in postmortem PD brains [23]. Our current studies show that a similar state of tauopathy also exists in a well-studied animal model of PD, the A53T α-Syn mutant mouse. Moreover, we also show that the changes in the tauopathy-associated proteins, α-Syn, p-Tau and p-GSK-3β, in the A53T mutant are similar to those which occur in postmortem PD striata. In the present study we have used the hemizygous A53T mouse, rather than the homozygous mouse, which has reduced severity and delayed onset of the disease. Moreover, we also used the 8 month old animal which is not yet symptomatic and in which the parkinsonism is also known to be less severe than in older animals. We nonetheless detected large and robust increases in p-Tau levels, hyperphosphorylated at Ser202, Ser262 and Ser396/404, which are the same major sites of hyperphosphorylation seen in the pathology of AD [35], [36]. In addition, we also observed large increases in p-GSK-3β, the major kinase known to hyperphosphorylate Tau at these epitopes.

Only limited neurochemical studies have been conducted demonstrating and identifying the epitopes of hyperphosphorylated Tau in postmortem tissues in PD brains or in animal models of PD. A recent study found increased levels of Tau hyperphosphorylated at Ser396 in synaptic-enriched fractions isolated from the frontal cortex of PD patients [37]. In animal models of PD, tauopathic changes have been observed in brainstem of symptomatic A30P α-Syn mutant mice at pSer202 and pSer396/404 [38]. Another study using the neurotoxin rotenone to model parkinsonism in rats, found immunoreactive Tau hyperphosphorylated at Ser202/Thr205, Thr212/Ser214 and Ser396/404 [39].

An interesting aspect of our studies is that the overexpressed A53T α-Syn seen in these animals was present as aggregates, forming dimmers and trimers of the monomeric 17 kDa and the 12 kDa polypeptides in the Triton X 100-insoluble fractions, as compared to wild-type animals. In addition, pSer396/404 Tau and p-GSK-3β were also found to be present in the Triton X-100-insoluble fractions, suggestive of their co-seggregation with α-Syn aggregates. Indeed, in previous studies [27], we have demonstrated that α-Syn, pSer396/404 Tau and p-GSK-3β interact with each other and are present in a trimeric complex, through stable protein:protein interactions with one another. Although Tau and α-Syn have distinct biological functions, *in vitro* studies have shown that α-Syn can induce fibrillization of Tau, and vice versa, with the A53T mutation accelerating and enhancing formation of fibrils as compared to wildtype α-Syn [12].

In the present study, we also found enhanced levels of pSer396/404 in cytoskeleton-free fractions, along with increased levels of p-GSK-3β. It is now well established that upon hyperphosphorylation, p-Tau dissociates from the microtubules [35], [36], and our results are consistent with this. The dissociation of Tau from microtubules leads to their destabilization and eventual collapse, resulting in neurodegeneration of neurons. Thus, in a mouse model of AD, neurons expressing Tau hyperphosphorylated at Ser202/Thr205 were more prone to undergo degeneration [40]. Hyperphosphorylation at both Ser262 and Ser396/404 has been shown to lead to reduced binding of Tau to microtubules resulting in increased destabilization of the microtubules [41] and, ultimately the collapse of the cytoskeleton. The scenario presented by Tau hyperphosphorylated at Ser202, Ser262 and Ser396/404 in the A53T α-Syn mutant is consistent with the degenerative changes observed in dopaminergic neurons innervating the striatum in PD, which we have shown earlier in a previous study [24]. Indeed, our results show that upon hyperphosphorylation, all three epitopes of p-Tau are exclusively present in the PIPES microtubule-free fraction [see Fig. 4B]. Upon further aging of these mice, or in the homozygous mouse where the disease is more severe, it is likely that microtubule destabilization results.

An additional aspect of our results that is worth mentioning is our findings regarding protein phosphatases. Thus, in neither the A53T mice nor in human PD brains were we able to find changes in PP1, PP2A or PP2B, suggesting that p-Tau formation were not linked to protein phosphtases. These findings are interesting, since decreases in protein phosphatases could have lead to increases in p-Tau levels.

We have also demonstrated here an increase in levels of p-GSK-3β, autophosphorylated at Tyr216, which represents the activated state of the kinase. GSK-3β is a proline-directed kinase which has been shown to hyperphosphorylate Tau at several known sites, including Ser202, Ser262 and Ser396/404 [42]–[45]. In neurons, GSK-3β's activity is inhibited by N-terminal phosphorylation at Ser9. Autophosphorylation at Tyr216 in GSK-3β acts in opposition to this by increasing GSK-3β kinase activity. In our study, p-GSK-3β activation mirrors increases in p-Tau levels in the striatum of both animals and humans, suggesting that this enzyme may be important in the genesis and maintenance of PD in humans. Indeed, a recent study demonstrated a strong linkage of two single nucleotide polymorphisms in the GSK-3β gene to sporadic PD [46]. Moreover, in previous studies, we have found that α-Syn can recruit p-GSK-3β through a direct physical protein:protein interaction to mediate the hyperphosphorylation of Tau, and that activation of p-GSK-3β by α-Syn precedes formation of p-Tau *in vitro* [27]. Our data also shows an increase in GSK-3β-Ser9, which represents the inactive form of the kinase. Interestingly, our findings showing increases in both p-GSK-3β and GSK-3β-Ser9 are similar to that seen reccently in Alzheimer's disease, where GSK3β was found to be phosphorylated to a similar extent at both the regulatory sites, Ser9 and Tyr216 [47].

The current study also demonstrates *in vivo* co-localization and accumulation of α-Syn, p-Tau and p-GSK-3β into aggregates, as evidenced from immunohistochemical staining of striatal slices from A53T mice. Moreover, we also observed a conformational change in p-Tau, defined by antibody MC1, representing the transition of p-Tau from soluble to aggregated filamentous Tau. The folding of Tau into a paperclip-like structure, recognized by MC1 antibodies, occurs when the amino acids at residues 7–9 interact with residues 312–342, and is one of the earliest pathological alterations of Tau in Alzheimer disease, preceding the formation of neurofibrillary tangles in AD [48]. Interestingly, the MC1 conformation is induced by hyperphosphorylation of Tau at Ser199, Ser202, Thr205 and the PHF-1 sites [Ser396/404], causing compaction of the paperclip structure [48]. Moreover, it has been shown that paperclip conformation of p-Tau enhances its ability to aggregate to paired helical filaments [48]. The presence of the aforementioned co-localized proteins *in-vivo* and the observation of greater amounts of the aggregated filamentous Tau seen in the A53T striatum also strengthens the conclusion that such interactions or aggregations are indeed pathogenic. The presence of neurotoxic aggregation correlates with the loss of TH positive neurons in the striatum when comparing A53T to wild type mice.

In conclusion, the present findings suggest that changes in Tau metabolism may indeed be a common denominator in neurodegenerative diseases such as PD and AD. Despite differences in their topographic distributions and phenotypic manifestations, these diseases are linked by the progressive accumulation of hyperphosphorylated Tau, activated GSK-3β and elevated levels of α-Syn. Taken together, these findings highlight the potential importance of targeting the tauopathic pathway in the therapeutic management of diverse human neurodegenerative diseases, including PD.

## Materials and Methods

### Materials

The antibodies used in this study are: anti-Tau MAB361 from Millipore [Temecula, CA]; anti-Tau Neurofibrillary Tangles Marker AHB0042 and anti-tau (pS262), Biosource Invitrogen [Carlsbad, CA]; anti-α-Syn CAT# 610787, anti-GSK-3β CAT# 612313 and anti-pGSK-3B [purified mouse anti-GSK-3β (pY216) CAT # 612313], from BD Transduction Labs [San Jose, CA]; anti-β-actin SC-1616, PP2B-Aα (C-20) SC-6123p-α-Syn (Ser129) SC-135638 and PP1 (E-9) SC-7482 from Santa Cruz Biotechnology, Inc. [Santa Cruz, CA]; The CP-13, PHF-1 and MC1 antibodies [recognizing Tau-Ser202, Tau-Ser396/404 and conformational-sensitive antibody, respectively] were gifts from Dr. Peter Davies [New York]; anti-α-Tubulin T6074 from Sigma Aldrich [St. Louis, MO]; PP2A 2039 and pGSK-3β (Ser9) 9336 were from Cell Signaling Technology (Danvers, Massachusetts); human p-α-Syn - Catalog #014-20281, Wako Chemicals USA, Inc., Richmond, VA; mouse anti-Tyrosine Hydroxylase Alexa Fluor 488 Conjugated Monoclonal MAB5280X from Chemicon International [Billerica, MA]; rabbit polyclonal to MAP1 ab25954 from Abcam Inc. [Cambridge, MA].

### Animals

Mice used in accordance with the Georgetown University Animal Care and Use Committee approval were bred and maintained in University-approved animal facilities and handled by trained personnel. Mice were group housed (2–5 animals/cage) in temperature- and humidity-controlled rooms under 12 h light/dark cycles and fed an ad-libitum diet of standard mouse chow. Mice were originally obtained in breeding pairs from Jackson laboratories (Bar Harbor, MA) to generate a stable breeding colony. A breeding colony of transgenic mice carrying the hA53T mutation driven by the mouse prion promoter was established according to previously characterized mice [28]. Mice hemizygous for the hA53T mutation were bred on a mixed C57BL/6J x C3H/HeJ background to produce transgenic and non-transgenic litter mates. In all cases, 2, 4 and 8 month-old transgenic mice were directly compared with age-matched non-transgenic (wild-type) litter mates. To identify transgenic mice, PCR amplifications were performed on 1 µL of proteinase K digested (Promega, Madison, WI) tail DNA samples using a sense (5′-TGTAGGCTCCAAAACCAAGG-3′) and an anti-sense primer (5′-TGTCAGGATCCACAGGCATA-3′). PCR reactions (30 µL) consisted of 10x NH_4_ buffer (Bioline, London, UK), 1.5 mM MgCl_2_, 0.05 mM each dNTP, 0.4 pM of each primer, and 0.1 units of *Taq* DNA polymerase (Bioline). Reactions were denatured at 95°C for 5 minutes and then subjected to 35 cycles of 95°C for 45 seconds, 61°C for 1 minute, 72°C for 1 minute, and 72°C for 2 minutes. A 30 µL aliquot from each reaction was analyzed by gel electrophoresis in a 1.5% agarose gel for the presence of a 248-base pair band.

### Postmortem tissue

Postmortem tissue was provided by the Sun Health Research Institute Brain donation program (Sun City, AZ) and included samples from PD cases that, antemortem, showed no evidence of dementia (and neuropathologically confirmed to be absent of AD pathology or cortical Lewy Bodies) and well characterized, neurologically/neuropathologically normal controls. Clinical evaluation and neuropathological diagnoses of these cases have been published in greater detail elsewhere [29]. The average postmortem interval was ∼3 hours. Data in this study were as follows: PD patients: 4 male and 2 female, ages 84–90, with mean age of 85.3 years; control group, 3 males and 3 females, ages 78–89, with mean age of 80.7 years. Since no gender differences were observed, data were pooled together.

Tissues were homogenized in buffer containing 80 mM PIPES (pH 6.8), 1 mM MgCl_2_, 2 mM EGTA. 0.1 mM EDTA, 0.1% Triton X-100 and 30% glycerol. Lysates were incubated at 37°C for 10 minutes prior to room temperature centrifugation at 14,000×g to separate soluble and insoluble fractions. Insoluble fractions were re-suspended in 2% SDS, 5 mM EDTA, 5 mM EGTA, 10% glycerol, 0.25 M Tris-HCl (pH 6.8) and sonicated with a Branson Sonifier. The fractions were combined to obtain total lysates and diluted with Laemmli buffer containing 5% β-mercaptoethanol, 5% SDS and 1% sodium deoxycholate. Samples were heated at 65°C for 1 hour and run on 10–20% Tris HCl Criterion gels (Bio-Rad).

### Preparation of Triton X-100 soluble and insoluble fractions

The aggregation state of α-Syn was analyzed based on its differential solubility in 1% Triton X-100, as described elsewhere [30]. Briefly, tissues were extracted in buffer containing 20 mM Tris-HCl, pH 7.4, 50 mM NaCl, 1% Triton X-100, protease inhibitor cocktail tablets (Complete Mini, EDTA-free; Roche Diagnostics GmbH, Germany) and phosphatase inhibitors (Halt™ Protease Inhibitor Cocktail; Pierce). Lysates were incubated for 30 min on ice, followed by centrifugation at 15,000×g for 60 min at 4°C. The pellet and supernatant were collected as the Triton X-100-insoluble and soluble fractions, respectively. The Triton X-100-insoluble pellets were redissolved in the previously described lysis buffer containing 2% SDS [30].

### Extraction of tissues in PIPES-SDS buffer

To measure interaction of proteins with the cytoskeleton, mouse striata were differentially extracted and separated into cytoskeleton-free and cytoskeleton-associated fractions as described previously [23]. Briefly, striata were homogenized in prewarmed [37°C] extraction buffer (80 mM PIPES, pH 6.8, 1 mM MgCl2, 2 mM EGTA, 0.1 mM EDTA, 0.1% Triton X-100 and 30% glycerol) containing protease inhibitors (Complete Mini, EDTA-free; Roche Diagnostics GmbH, Germany) and phosphatase inhibitors (Halt™ Protease Inhibitor Cocktail; Pierce). After a 10 min incubation period at 37°C, lysates were centrifuged (20 min, 14,000×g) at room temperature and the supernatant removed; the supernatant contained soluble cytoskeletal-free proteins. The pellet, containing the cytoskeletal-associated proteins, was resuspended in 2% SDS, 5 mM EDTA, 10% glycerol, 0.25 mM Tris-HCl, pH 6.8 and sonicated with a Branson Sonifier 250.Sonication was done 3 times for 30 s at room temperature, with cooling between bursts. Proteins in both fractions were analyzed by Western blots.

### Western Blot Analysis

Western blot analysis was performed as described elsewhere [24], [27]. Briefly, mouse striata were homogenized in RIPA buffer (50 mM Tris-HCl pH 7.5, 150 mM NaCl, 1 mM EDTA) containing 0.5% Triton X-100, 0.5% sodium deoxycholate, and 0.1% SDS in the presence of protease inhibitor cocktail tablets (Complete Mini, EDTA-free; Roche Diagnostics GmbH, Germany) and phosphatase inhibitor cocktail (Halt™ Protease Inhibitor Cocktail; Pierce). Lysates were left on ice for 20 minutes and sonicated for 30 seconds with a Branson Sonifier. Lysates were inverted at 4°C for 30 min, followed by centrifugation for 10 min at 14,000×g and 4°C. Supernatants were collected and protein concentrations were measured using the Lowry assay. Samples were analyzed by Western blots on 10–20% Tris-HCl Criterion gels (Bio-Rad), after blocking with 20 mM Tris-buffered saline, pH 7.6 containing 0.1% Tween 20 (TBST) and 5% (wt/vol) blotting grade blocker non-fat dry milk (Bio-Rad) for 1 hour at room temperature. Western blots were developed with a wide range of specific human Tau antibodies that recognize the protein at different phosphorylation sites, including: CP13 (pS202) (1∶500), PHF-1 (pS396/404) (1∶1000) and pS262 (1∶500). Total Glycogen Synthase Kinase-3β (GSK-3β) was probed for with mouse GSK-3β antibody (1∶500) and p-GSK-3β was probed for using mouse phospho-specific (pY216) antibody (1∶500). This antibody also recognizes p-GSK-3α, detectable as a band visible above p-GSK-3β; in our studies, only the lower band, corresponding to p-GSK-3β was used to calculate for this protein. To probe for α-Syn, samples were run on 10-20% Tris HCl Criterion gels (Bio-Rad) and immunoblotted with mouse α-synuclein (1∶500) antibody. All proteins were normalized to total Tau (1∶500) or β-actin. After incubation for 2 hours at room temperature with HRP-conjugated secondary antibodies (1∶3000; Santa Cruz), proteins were revealed by enhanced chemiluminescence (Perkin Elmer). Images were scanned by Scanner EPSON Perfection V700 Photo and then quantified using ImageJ.

### Immunohistochemistry

IHC analysis of mouse brain coronal sections was performed as previously described [24], with slight modifications. Briefly, mouse brains from 12 month old control wild-type and age-matched A53T α-Syn mutant mice were perfused with 4% PFA, and prepared in a sequential sucrose gradient, from 10% to a final 30% sucrose soak. Sections were washed, permeabilized, and stained in the following manner. Each slice was washed 3 times in 1 mg/ml NaBr_2_, 1 X PBS pH 7.4, for 5 min at room temperature. Following the NaBr_2_ auto-fluorescence quenching treatment, each slice was washed 6 X, for 10 min in 1 X PBS pH 7.4, 1% Triton X-100 followed by blocking for 1hr at room temperature in 1 X PBS pH 7.4, 1% Triton X-100, 10% FCS. Antibodies were conjugated primarily to the appropriate fluorophore as indicated using the following reagents and manufacturer's protocol: Lighting-Link FITC #707-0030, Lighting-Link Rhodamine #710-0030, Lighting-Link Texas Red #714-0030 (Novus Biologicals Littleton, CO). Incubation with primary antibody occurred at 4°C, overnight in the dark, in blocking buffer using the following concentrations for either single or dual staining with the following fluorophore conjugated antibodies: anti-α-Syn, Texas Red 1∶750; Anti-p-GSK-3β (pY216), Texas Red; Anti-p-GSK-3β (pY216), FITC 1∶500; PHF-1, FITC 1∶500; MC1, Rhodamine 1∶500. Following staining, each slice was washed 3 X in 1 X PBS pH 7.4, 1% Triton X-100 at room temperature, incubated for 30 min in blocking buffer, and washed a final 3X in 1X PBS pH 7.4, 1% Triton X-100. Stained slices were mounted to Fischer Scientific Superfrost standard microscope slides using Fluoromount-DAPI. Fluorescence images were captured using a laser scanning confocal microscope (Olympus FV300). Paired images between WT and Tg tissue for all figures were collected at the same laser power, gain, and offset settings. Postcollection processing was performed using ImageJ and applied uniformly to all paired images.

### Statistical Analysis

Results were expressed as mean ± S.E.M. and statistically analyzed by the Student's t test between two groups and analysis of variance among multiple groups. Statistical significance was accepted at the [*P<*0.05] level.

## References

[1] Sidhu A, Wersinger C, Moussa CE, Vernier P (2004). The role of alpha-synuclein in both neuroprotection and neurodegeneration.. Ann N Y Acad Sci.

[2] Hofer A, Berg D, Asmus F, Niwar M, Ransmayr G (2005). The role of alpha-synuclein in gene multiplications in early-onset Parkinson's disease and dementia with Lewy bodies.. J Neural Transm.

[3] Kruger R, Kuhn W, Muller T, Woitalla D, Graeber M (1998). Ala30Pro mutation in the gene encoding alpha-synuclein in Parkinson's disease.. Nat Genet.

[4] Polymeropoulos MH, Lavedan C, Leroy E, Ide SE, Dehejia A (1997). Mutation in the alpha-synuclein gene identified in families with Parkinson's disease.. Science.

[5] Goedert M, Spillantini MG (1998). Lewy body diseases and multiple system atrophy as alpha-synucleinopathies.. Mol Psychiatry.

[6] Goedert M, Spillantini MG, Davies SW (1998). Filamentous nerve cell inclusions in neurodegenerative diseases.. Curr Opin Neurobiol.

[7] Arai Y, Yamazaki M, Mori O, Muramatsu H, Asano G (2001). Alpha synuclein-positive structures in cases with sporadic Alzheimer's disease: morphology and its relationship to tau aggregation.. Brain Research.

[8] Ishizawa T, Mattila P, Davies P, Wang D, Dickson DW (2003). Colocalization of tau and alpha-synuclein epitopes in Lewy bodies.. J Neuropathol Exp Neurol.

[9] Tsuboi Y, Wszolek ZK, Graff-Radford NR, Cookson N, Dickson DW (2003). Tau pathology in the olfactory bulb correlates with Braak stage, Lewy body pathology and apolipoprotein epsilon4.. Neuropathol Appl Neurobiol.

[10] Duda JE, Giasson BI, Mabon ME, Miller DC, Golbe LI (2002). Concurrence of alpha-synuclein and tau brain pathology in the Contursi kindred.. Acta Neuropathol.

[11] Yamaguchi K, Cochran EJ, Murrell JR, Polymeropoulos MH, Shannon KM (2005). Abundant neuritic inclusions and microvacuolar changes in a case of diffuse Lewy body disease with the A53T mutation in the alpha-synuclein gene.. Acta Neuropathol.

[12] Kotzbauer PT, Giasson BI, Kravitz AV, Golbe LI, Mark MH (2004). Fibrillization of alpha-synuclein and tau in familial Parkinson's disease caused by the A53T alpha-synuclein mutation.. Exp Neurol.

[13] Yamazaki M, Arai Y, Baba M, Iwatsubo T, Mori O (2000). Alpha-synuclein inclusions in amygdala in the brains of patients with the parkinsonism-dementia complex of Guam.. J Neuropathol Exp Neurol.

[14] Forman MS, Schmidt ML, Kasturi S, Perl DP, Lee VM (2002). Tau and alpha-synuclein pathology in amygdala of Parkinsonism-dementia complex patients of Guam.. Am J Pathol.

[15] Hamilton RL (2000). Lewy bodies in Alzheimer's disease: a neuropathological review of 145 cases using alpha-synuclein immunohistochemistry.. Brain Pathol.

[16] Jellinger KA (2004). Lewy body-related alpha-synucleinopathy in the aged human brain.. J Neural Transm.

[17] Nemes Z, Devreese B, Steinert PM, Van Beeumen J, Fesus L (2004). Cross-linking of ubiquitin, HSP27, parkin, and alpha-synuclein by gamma-glutamyl-epsilon-lysine bonds in Alzheimer's neurofibrillary tangles.. FASEB J.

[18] Griffin WS, Liu L, Li Y, Mrak RE, Barger SW (2006). Interleukin-1 mediates Alzheimer and Lewy body pathologies.. J Neuroinflammation.

[19] Szpak GM, Lewandowska E, Lechowicz W, Bertrand E, Wierzba-Bobrowicz T (2001). Lewy body variant of Alzheimer's disease and Alzheimer's disease: a comparative immunohistochemical study.. Folia Neuropathol.

[20] Lei P, Ayton S, Finkelstein DI, Adlard PA, Masters CL (2010). Tau protein:. Relevance to Parkinson's disease.. Int J Biochem Cell Biol 2010 Aug 1.

[21] Lippa CF, Fujiwara H, Mann DM, Giasson B, Baba M (1998). Lewy bodies contain altered alpha-synuclein in brains of many familial Alzheimer's disease patients with mutations in presenilin and amyloid precursor protein genes.. Am J Pathol.

[22] Iseki E, Marui W, Kosaka K, Ueda K (1999). Frequent coexistence of Lewy bodies and neurofibrillary tangles in the same neurons of patients with diffuse Lewy body disease.. Neurosci Lett.

[23] Wills J, Jones J, Haggerty T, Valeriy D, Joyce J (2010). Elevated tauopathy and alpha-synuclein pathology in postmortem Parkinson's disease brain with and without dementia.. Exp Neurol.

[24] Duka T, Rusanak M, Drolet R, Duka V, Wersinger C (2006). Alpha-synuclein induces hyperphosphorylation of Tau in the MPTP model of Parkinsonism.. FASEB J.

[25] Duka T, Sidhu A (2006). The Neurotoxin MPP induces hyperphosphorylation of Tau in the presence of alpha-synuclein in SHSY-5Y neuroblastoma cells.. Neurotox Res.

[26] Kozikowski AP, Gaisina IN, Petukhov PA, Sridhar J, King LT (2006). Highly potent and specific GSK-3beta inhibitors that block tau phosphorylation and decrease alpha-synuclein protein expression in a cellular model of Parkinson's disease.. ChemMedChem.

[27] Duka T, Duka V, Joyce JN, Sidhu A (2009). Alpha-Synuclein contributes to GSK-3beta-catalyzed Tau phosphorylation in Parkinson's disease models.. FASEB J.

[28] Giasson BI, Duda JE, Quinn SM, Zhang B, Trojanowski JQ (2002). Neuronal alpha-synucleinopathy with severe movement disorder in mice expressing A53T human alpha-synuclein.. Neuron.

[29] Joyce JN, Ryoo HL, Beach TB, Caviness JN, Stacy M (2002). Loss of response to levodopa in Parkinson's disease and co-occurrence with dementia: role of D3 and not D2 receptors.. Brain Res.

[30] Zhou W, Freed CR (2004). Tyrosine-to-cysteine modification of human alpha-synuclein enhances protein aggregation and cellular toxicity.. J Biol Chem.

[31] Jeannotte AM, Sidhu A (2008). Regulated interactions of the norepinephrine transporter by actin and microtubule cytoskeletons.. J Neurochem.

[32] McCormack AL, Atienza JG, Johnston LC, Andersen JK, Vu S (2005). Role of oxidative stress in paraquat-induced dopaminergic cell degeneration.. J Neurochem.

[33] Kramer ER, Aron L, Ramakers GM, Seitz S, Zhuang X (2007). Absence of Ret signaling in mice causes progressive and late degeneration of the nigrostriatal system.. PLoS Biol.

[34] Barroso-Chinea P, Cruz-Muros I, Aymerich MS, Rodríguez Díaz M, Afonso-Oramas D (2005). Striatal expression of GDNF and differential vulnerability of midbrain dopaminergic cells.. Eur J Neurosci.

[35] Abraha A, Ghoshal N, Gamblin TC, Cryns V, Berry RW (2000). C-terminal inhibition of Tau assembly in vitro and in Alzheimer's disease.. Cell Sci.

[36] Weaver CL, Espinoza M, Kress Y, Davies P (2000). Conformational change as one of the earliest alterations of Tau in Alzheimer's disease.. Neurobiol Aging.

[37] Muntané G, Dalfó E, Martinez A, Ferrer I (2008). Phosphorylation of Tau and alpha-synuclein in synaptic-enriched fractions of the frontal cortex in Alzheimer's disease, and in Parkinson's disease and related alpha-synucleinopathies.. Neuroscience.

[38] Frasier M, Walzer M, McCarthy L, Magnuson D, Lee JM (2005). Tau phosphorylation increases in symptomatic mice overexpressing A30P alpha-synuclein.. Exp Neurol.

[39] Höglinger GU, Lannuzel A, Khondiker ME, Michel PP, Duyckaerts C (2005). The mitochondrial complex I inhibitor rotenone triggers a cerebral Tauopathy.. J Neurochem.

[40] Schindowski K, Bretteville A, Leroy K, Begard S, Brion JP (2006). Alzheimer's disease-like tau neuropathology leads to memory deficits and loss of functional synapses in a novel mutated tau transgenic mouse without any motor deficits.. Am J Pathol.

[41] Zhong J, Iqbal K, Grundke-Iqbal I (1999). Hyperphosphorylated tau in SY5Y cells: similarities and dissimilarities to abnormally hyperphosphorylated tau from Alzheimer disease brain.. FEBS Lett.

[42] Utton MA, Vandecandelaere A, Wagner U, Reynolds CH, Gibb GM (1997). Phosphorylation of tau by glycogen synthase kinase 3beta affects the ability of tau to promote microtubule self assembly.. Biochem J.

[43] Sengupta A, Kabat J, Novak M, Wu Q, Grundke-Iqbal I (1998). Phosphorylation of tau at both Thr 231 and Ser 262 is required for maximal inhibition of its binding to microtubules.. Arch of Biochem Biophys.

[44] Liu SJ, Zhang JY, Li HL, Fang ZY, Wang Q (2004). Tau becomes a more favorable substrate for GSK-3 when it is prephosphorylated by PKA in rat brain.. J Bio Chem.

[45] Liu F, Li B, Tung EJ, Grundke-Iqbal I, Iqbal K (2007). Site-specific effects of tau phosphorylation on its microtubule assembly activity and self-aggregation.. Eur J Neurosci.

[46] Kwok JB, Hallupp M, Loy CT, Chan DK, Woo J (2005). GSK3B polymorphisms alter transcription and splicing in Parkinson's disease.. Ann Neurol.

[47] Soutar MP, Kim WY, Williamson R, Peggie M, Hastie CJ (2010). Evidence that glycogen synthase kinase-3 isoforms have distinct substrate preference in the brain.. J Neurochem Nov.

[48] Jeganathan S, Hascher A, Chinnathambi S, Biernat J, Mandelkow EM (2008). Proline-directed pseudo-phosphorylation at AT8 and PHF1 epitopes induces a compaction of the paperclip folding of Tau and generates a pathological (MC-1) conformation..

